# Supplementary material for: Evaluation of the relationship between consumption of carbonated soft drinks/fast food and anxiety-related sleep disturbance in school adolescents in Bangladesh
Source: PLOS Glob Public Health. 2025 Mar 10;5(3):e0004322. doi: 10.1371/journal.pgph.0004322 (PMC11892876; doi:10.1371/journal.pgph.0004322)
Supplement: Attachment — Submitted filename: Response to Reviewer(s).docx [file pgph.0004322.s001.docx]

December 31, 2024

Feten Fekih-Romdhane
Academic Editor
PLOS Global Public Health

**Re: Manuscript Number PGPH-D-24-01105R1**

Dear Dr. Feten Fekih-Romdhane,

Thank you for considering and giving us the chance to submit the revision of our manuscript, **Consumption of carbonated soft drinks and fast food, and sleep disturbance associated with anxiety among school-going adolescents in Bangladesh,** for possible publication in PLOS Global Public Health. We revised the manuscript based on the reviewers feedback. Their useful and productive comments helped us improve the clarity and quality of the manuscript. Where we changed the text, the corresponding sentences have been highlighted in track changes. Where textual changes are included in this response to the reviewer, we have indented and italicized them and removed references.

We hope that the revisions are satisfactory in addressing issues raised by the reviewers, and we look forward to hearing your opinion about this article.

Yours sincerely,

Corresponding Author

**Comments to the Author**

**Reviewer #1:** The paper is well written in an easily comprehensible manner. However, I have the following concerns:

1. Given that the data upon which your discussion is based is a decade old, what limitations might this pose to the study? Your response to this could be included in the caveat section that you already discussed in the paper.

**Response:** Thank you very much for your thoughtful observation and suggestion. This will surely enhance the clarity of the findings. We have addressed this issue in the caveat section as follows:

*“**Finally, the most recent GSHS in Bangladesh was completed in 2014; hence we lack new evidence regarding the correlation between unhealthy food patterns and sleep disturbances. Evidence indicates a rising consumption of fast food and soft drinks among youth, and its correlation with anxiety-related sleep disturbances aligns with prior global research, underscoring the significance of this study's findings.”*

2. The p-values in your Table 3 shows that the association of many of your independent variables (including the soft drinks and fast food variables) with the dependent variable (anxiety related sleep loss) is not statistically significant. How may this affect the interpretation of the associations you have provided in the results section?

**Response:** Thank you. Yes, we did not find a significant relationship between many dependent variables, including the consumption of soft drinks and fast food; that’s why we did not present the findings of insignificant results. However, since the consumption of soft drinks and fast food were the main exposure variables of interest, and the prevalence of anxiety-related sleep disturbance was higher among adolescents who consume soft drinks and fast food, therefore we present those findings in the result section, though they are not significant.

**Reviewer #2:** General comments
1)The authors have selected an interesting topic pertaining to adolescent health. Here are few recommendations.

**Response:** Thank you very much for your appreciation. In the following sections we presented how we accommodated your valuable recommendations in the revised manuscript.

2) The article requires english grammar correction, as few sentences were difficult to comprehend.

**Response:** Thank you. We have thoroughly checked the manuscript and corrected the grammatical mistakes as well as rewritten some sentences for better clarification.

Abstract:
In the results section of abstract it would be better to not comment on bullying victimization, substance use and other factors on the effect of sleep. As the main objective focuses on association between consumption of carbonated drinks, fast food on anxiety related sleep loss

**Response:** Thank you very much. As per your recommendation, we have removed the sentences that present the results for factors other than the consumption of fast food and soft drinks from the abstract.

Introduction:
1) The authors can provide global statistics pertaining to anxiety related sleep loss among adolescents.

**Response:** Thank you. We have added the following sentences in the first paragraph of the introduction section of the revised manuscript.

*“Globally, 36% of adolescents have been shown to encounter sleep issues. A recent systematic study and meta-analysis indicated that the total pooled prevalence of sleep problems among Chinese adolescents is 26%. Another cohort study in Germany involving adolescents aged 10–17 years revealed that over 20% of individuals experienced sleep-related issues. However, a multi-country study utilizing the most recent data from the Global School-Based Health Survey indicated that the pooled weighted prevalence of anxiety-induced sleep disturbance was 10.7%.”*

2) As authors have also find association between various factors and anxiety related sleep loss it would be better to add it as secondary objective in the concluding section of introduction.

**Response:** Thank you for your thoughtful suggestion. Since our focus was to assess the relationship between the consumption of fast food and soft drinks and anxiety-related sleep loss, we wanted to concentrate on this relationship. Other variables were included as covariates. Including those factors as secondary objectives would be better but may deviate from the main focus. Therefore, we concentrate only on our main objective and discuss that.

Methodology:
1)In the first line citing GSHS, the authors can provide reference for the survey.

**Response:** Thank you. We have provided a reference according to your suggestion in the revised version.

2) Authors have to mention the study period of secondary data analysis of GSHS data.

**Response:** Thank you. We have added it in the first line of the method section in revised manuscript.

Results:
1) The authors can omit Table 1.

**Response:** Thank you. We have provided Table 1 for better understanding the way the variables were coded. Otherwise, we have to write in detail how we categorize each of the variables. We think Table 1 helps readers more clearly understand the definition and categorization of the variables.

Discussion:
1) Discussion looks fragmented. It would be better for the authors to write discussion in sequence aligned with results presentation.

**Response:** Thank you for your suggestion. We have revised and rewritten the discussion section as per your suggestion and suggestions by other reviewers.

**Reviewer #3:** The study addresses a pertinent issue in adolescent health—dietary habits and their impact on mental health and sleep quality, which is highly relevant, especially in developing countries like Bangladesh where these trends are under-researched. The manuscript is well-structured, with a clear flow from the abstract to conclusions.
However, there are some minor changes that can be made to this manuscript.

**Response:** Thank you very much for your kind words of appreciation. The following sections explain how we addressed your concerns.

The abstract can be made more concise by shortening the sentences that discuss odds ratios (ORs) could be shortened.

**Response:** Thank you. We have rewritten the specific sentence in the revised version according to your suggestion and following the suggestion of reviewer 4.

Also, the prevalence of hunger, bullying, and other factors that were included could be briefly mentioned without going into too much detail.

**Response:** Thank you. We did not include the prevalence of those factors in the abstract because our study's goal was solely to establish a link between adolescents' junk food habits and anxiety-related sleep loss. Additionally, reviewer 1, we suggested removing the results of other factors from the abstract. Therefore, we have deleted results of the covariates included in the study.

In the introduction, the manuscript could benefit from a more focused research question at the end of the introduction, explicitly stating what gap this study addresses in the context of Bangladesh.

**Response:** Thank you. We have added the following sentence at the end of the introduction section.

*“It is crucial to address this area of research in Bangladesh, where resources to meet adolescents' mental health needs are scarce, and there is evidence that the consumption of fast food and soft drinks is rising among young generations, in order to develop effective intervention strategies.”*

In the methodology, clarify some of the operational variables such as "food insecurity" and "physical violence" which can be described in detail or in reference to previous studies that have used these specific measures.

**Response:** Thank you. In Table 1, we have presented a complete list of covariates used in this current analysis, with their categories and coding. The coding used in this analysis was followed by previous studies that used the Global School-based Health Survey dataset, and we also provided the references in the revised version.

The results section is well-explained, but some parts are over-detailed. For example, the specific prevalence rates of hunger and other variables in Table 2 could be streamlined or moved to supplementary material.

**Response:** Thank you. Table 2 delineates the background features, facilitating a clear comprehension of the sample for readers. Consequently, we have reported the results of several variables here.

It might also help to include a more detailed breakdown of how fast food and soft drink consumption vary by demographic characteristics like age and gender.

**Response:** Thank you very much for your thoughtful suggestion. Analysis stratified by age and gender would be better; we did run the analyses stratified by age and sex. However, while stratified by age and gender, we did not find enough samples for categories of many variables. This might be due to the low prevalence of anxiety-related sleep loss in this sample. Therefore, we did not present stratified analysis. 

The discussion effectively compares findings to previous studies but could be enhanced by integrating a more thorough exploration of cultural or socioeconomic factors unique to Bangladesh that might explain the results.
The discussion on the potential biological mechanisms of how fast food and soft drinks might contribute to anxiety-related sleep is valuable but should also consider psychosocial mechanisms such as stress related to unhealthy dietary habits.

**Response:** I sincerely appreciate it. In the revised manuscript, we have included two additional paragraphs that provide an explanation of the socioeconomic and psychosocial factors that elucidate the connection between poor food habits and sleep loss.

*“Adolescents demonstrate a significantly increased risk for insufficient sleep duration. Multiple specific behavioral risk factors may account for this finding, such as increased electronic device usage and suboptimal dietary habits. A systematic review of studies has shown that the consumption of junk foods, including ultra-processed foods, fast foods, unhealthy snacks, and sugar-sweetened beverages, increases the risk of stress and depression. Sleep disturbances, including insomnia, narcolepsy, sleep-disordered breathing, and restless legs syndrome (RLS), occur in about 90% of individuals with depression. Moreover, adolescence represents a critical transitional phase characterized by elevated stress levels, significant social adjustments, and heightened sensitivity to stress. Research has shown a strong correlation between childhood trauma and current stressful life events with sleep disruptions, even when controlling for depression and anxiety.”*

*‘The increasing prevalence and accessibility of social media in developing nations like Bangladesh, especially among the youth, presents notable concerns regarding its frequent utilization by adolescents. The increase in nighttime use of electronic devices, late-night social activities, substance consumption, fast food intake, and caffeinated drinks, along with early school start times, leads to a greater prevalence of insufficient sleep or reduced sleep quality among adolescents. This usage also correlates with anxiety, stress, depression, reduced mood, and low self-esteem, along with decreased sleep duration and increased sleep disturbances in this population. Furthermore, adolescence is a critical developmental stage where individuals gain autonomy, enhance social skills, and develop a strong tendency to adopt modern lifestyles. The demands placed by educational institutions for increased study commitments and homework significantly reduce sleep duration, resulting in fatigue, fewer opportunities for physical activity, and a greater reliance on energy-dense foods during prolonged wakefulness among young individuals. These social and behavioral factors considerably exacerbate previously hypothesized processes within the body, such as hormone dysregulation, metabolic pathway changes, and daytime drowsiness and weariness, which therefore exacerbate problems with sleep. Additionally, adolescents often represent a primary demographic for the marketing of energy-dense, nutrient-poor food and beverage products. An obesogenic environment in adolescents may lead to the development of eating behaviors and dietary choices that heighten the risk of sleep disturbances or sleep loss.”*

**Reviewer #4:** - In the title, specify "anxiety-related sleep loss" to "sleep disturbances associated with anxiety" for clarity.

**Response:**  Thank you very much. We have revised the title according to your suggestion.

- For abstract, mention the covariates used in the analysis to provide a more complete overview of the study's methodology.

**Response:** Thank you. We have added the covariates in the abstract.

- In the introduction, strengthen the transition between global findings and the Bangladesh-specific context, particularly including lifestyle changes due to urbanization.

**Response:** Thank you. We have added the following sentences in the introduction section of the revised manuscript.

*“Moreover, historically it is evident that industrialization in high-income countries resulted in rural-to-urban migration and a shift in nutrition from predominantly plant-based foods—such as vegetables, fruits, whole grains, and legumes—to hyper-caloric diets characterized by high levels of total and saturated fats, cholesterol, animal protein, added salt, and sugar, while being low in fiber. Currently, various regions globally, including Bangladesh, are undergoing rapid urbanization, which may lead to socio-economic changes as well as shifts in lifestyle and dietary patterns from traditional diets to Western-like diets. This transition is accompanied increased access to a wider variety of foods, including high-calorie and processed options. The impact of lifestyle and dietary changes resulting from rapid urbanization in low- and middle-income countries, including Bangladesh, has accelerated the rising burden of non-communicable diseases.”* 

- Methods section, justify the inclusion of covariates in the logistic regression model by explaining their connection to anxiety-related sleep loss. Clarify how missing data were handled, what tests were done. Further explain why certain variables, such as substance use and physical violence, were included and how they were measured.

**Response:** Thank you. We have added references for justification of including variables in the analysis in the revised version of the manuscript.

Missing values were deleted on a case-by-case basis, as indicated in the methods section. Furthermore, due to the relatively low prevalence of anxiety-related sleep disturbances, the bivariate analysis revealed several cells with minimal responses (1 or 0). Consequently, we excluded certain variables, including substance abuse and sexual behavior, from the current analysis.

How we categorize variables is explained in Table 1; moreover, a detailed explanation is also given in the method section.

- Visuals.. for results, add visual representations (e.g., bar graphs) to show the distribution of fast food and soft drink consumption in relation to sleep loss for better clarity.

**Response:** Thank you. We have added Figure 2 in the revised version.

- In the results, provide a clearer explanation of how confounding variables influence the relationship between fast food, soft drinks, and anxiety-related sleep loss.

**Response:** Thank you. Since our aim was to assess the relationship between the consumption of soft drinks or fast food and anxiety-related sleep disturbances, therefore, we only focused on explaining the results of the variables of interest. Therefore, we did not explain the results of other covariates here.

- In the discussion, consider reverse causality—whether sleep loss leads to increased consumption of fast food and soft drinks. Also, expand on the limitations, particularly regarding the biases from self-reported data on sensitive topics like substance use and sexual behavior.

**Response:** Thank you. We found that consumption of soft drinks or fast food is associated with anxiety-related sleep disturbances; however, the nature of this study design prevents us from drawing the directionality. Either way, the relationship could be possible. Earlier studies also documented that sleep loss leads to increased soft drink and fast food consumption. Since our aim was to assess the relationship between the consumptions of soft drinks or fast food and anxiety-related sleep disturbances, we only focused on discussing this direction of the relationship.

- The conclusion is vague right now, include specific recommendations for policymakers and public health practitioners in Bangladesh to apply the findings.

**Response:** Thank you. We have revised the conclusion section and added the following sentences according to your suggestion.

***“****While the directionality of the relationship remains unknown, the results of this study indicate the potential benefits of implementing educational interventions for both parents and children via community- and school-based initiatives aimed at addressing unhealthy food consumption and its negative repercussions.”*

- In tables and figures, clarify labels and legends, especially for Figure 1, to improve data interpretation.

**Response:** Thank you. We have revised accordingly. Figure legends are provided after the references.

- In the references, ensure that all references are directly relevant to the findings and reduce reliance on studies from high-income countries without proper justification.

**Response:** Thank you. We have revised the manuscript accordingly.
