# Supplementary material for: Evaluation of the relationship between consumption of carbonated soft drinks/fast food and anxiety-related sleep disturbance in school adolescents in Bangladesh
Source: PLOS Glob Public Health. 2025 Mar 10;5(3):e0004322. doi: 10.1371/journal.pgph.0004322 (PMC11892876; doi:10.1371/journal.pgph.0004322)
Supplement: Attachment — Submitted filename: Response_to_Reviewer(s)_auresp_2.docx [file pgph.0004322.s002.docx]

January 21, 2025

Feten Fekih-Romdhane
Academic Editor
PLOS Global Public Health

**Re: Manuscript Number PGPH-D-24-01105R2**

Dear Dr. Feten Fekih-Romdhane,

Thank you for considering and giving us another chance to submit the revision of our manuscript, **Evaluation of the relationship between consumption of carbonated soft drinks/fast food and anxiety-related sleep disturbance in school adolescents in Bangladesh,** for possible publication in PLOS Global Public Health. We revised the manuscript based on the academic editor’s feedback. Where we changed the text, the corresponding sentences have been highlighted in track changes.

We hope that the revisions are satisfactory in addressing issues raised by the academic editor, and we look forward to hearing your opinion about this article.

Yours sincerely,

Corresponding Author

**Journal Requirements:**

Please review your reference list to ensure that it is complete and correct. If you have cited papers that have been retracted, please include the rationale for doing so in the manuscript text, or remove these references and replace them with relevant current references. Any changes to the reference list should be mentioned in the rebuttal letter that accompanies your revised manuscript. If you need to cite a retracted article, indicate the article’s retracted status in the References list and also include a citation and full reference for the retraction notice.

**Response:** We have checked all the references and those are correct. **Additional Editor Comments:

Before we can accept your paper for publication, the title should be changed to : "Evaluation of the relationship between consumption of carbonated soft drinks/fast food and anxiety-related sleep disturbance in school adolescents in Bangladesh."**

**Response:** We have changed the title accordingly.
